# Epidemiological investigation and antimicrobial susceptibility analysis of mycoplasma in patients with genital manifestations

**DOI:** 10.1002/jcla.23118

**Published:** 2019-11-12

**Authors:** Xiaodong Gu, Sudong Liu, Xuemin Guo, Ruiqiang Weng, Zhixiong Zhong

**Affiliations:** ^1^ Research Experimental Center Meizhou People's Hospital (Huangtang Hospital) Meizhou Hospital Affiliated to Sun Yat‐sen University Meizhou China; ^2^ Guangdong Provincial Key Laboratory of Precision Medicine and Clinical Translational Research of Hakka Population Meizhou China; ^3^ Guangdong Provincial Engineering and Technological Research Center for Molecular Diagnostics of Cardiovascular Diseases Meizhou China; ^4^ Center for Cardiovascular Diseases Meizhou People's Hospital (Huangtang Hospital) Meizhou Hospital Affiliated to Sun Yat‐sen University Meizhou China

**Keywords:** antimicrobial susceptibility, Hakka, infection, mycoplasma

## Abstract

**Background:**

The aim of this study was to investigate the infection and antimicrobial resistance of *Ureaplasma urealyticum* and *Mycoplasma hominis* in patients with genitourinary symptoms among Hakka population in Meizhou, China.

**Methods:**

A total of 12 633 females and 3315 males who presented urogenital symptoms and were subjected to mycoplasma tests from 2014 to 2018 were enrolled in this study. The mycoplasma detection and antimicrobial susceptibility were tested using the Mycoplasma ID/AST kit.

**Results:**

The total incidence of mycoplasma infection, as well as the incidence of *U urealyticum* in Hakka population was annually increasing from 2014 to 2018. The total incidences and *U urealyticum* infection were more prevalent in females than males. Higher positive rate of mycoplasmas infection was observed in women aged 16‐20 (50.9%) and men aged 26‐30 (25.4%). The occurrence of antimicrobial resistance of mycoplasma to antibacterial agents remained relatively similar in the past five years. *Ureaplasma urealyticum* infection, *M hominis* infection, and co‐infection of resistance to levofloxacin, erythromycin, ciprofloxacin, ofloxacin, roxithromycin, azithromycin, clarithromycin, and sparfloxacin were dramatically higher in females than in males.

**Conclusion:**

Our findings indicate a high burden of mycoplasmas infection and antimicrobial resistance of mycoplasmas infection among females, and josamycin and minocycline may be recommended as the primary choice in clinical treatment of anti‐mycoplasmas.

## 1 INTRODUCTION

Mycoplasma is known as the smallest free‐living organisms without cell wall. Mycoplasma including *Ureaplasma urealyticum* (*Uu*) and *Mycoplasma hominis* (*Mh*) causes genital manifestations in both women and men.^1^ It was estimated that 40%‐80% of healthy adult female infected with *Uu* and 20%‐50% with *Mh* in cervix or vagina.^2^, ^3^ Meanwhile, *Uu* infects 20%‐29% healthy males in urogenital tract.^4^ Previous study reported that genital mycoplasmas were related to urogenital infections, including urethritis, vaginitis, cervicitis, and pelvic inflammatory disease.^5^ However, it is difficult to prove their pathogenic effect because of their existence in genital tract of healthy human. The prevalence of these organisms is significantly associated with age, socioeconomic status, physiological cycle, pregnancy, and multiple sex partners.^6^, ^7^

As *Uu* and *Mh* lack of peptidoglycan, β‐lactams are completely inactive against them. Generally, quinolones, tetracyclines, and macrolides are used for the treatment of mycoplasma infection.^8^ However, drug resistance of mycoplasma increased due to the improper use of the antibiotics. Since antibiotic resistance of many pathogens is continually changing, surveillance studies are required for assisting in the optimization of antimicrobial treatment.

The city of Meizhou, located in southern China, is known as the world's Hakka population capital, with unique population‐based culture and food, as well as different physiological characteristics.^9^ However, little was known regarding the prevalence and antimicrobial susceptibility of mycoplasma infection in Hakka population.

The purpose of this study was to determine the prevalence and antimicrobial resistance of *Uu* and *Mh* in patients of Hakka population and presented genital manifestations. Our study would provide useful information for local epidemiology of mycoplasma, and thus help make the prevention and treatment strategy.

## 2 MATERIALS AND METHODS

### 2.1 Patients

A total of 15 948 patients committing mycoplasma examination from April 15, 2014, to December 31, 2018, in the clinical laboratory of Meizhou People's Hospital were enrolled in this study. Eligible patients should meet four criterions: (a) native Hakka who lived in Meizhou for more than three generations; (b) age 16‐89; (c) with suspected symptoms of genital tract infection (including burning sensation or pain during urination, difficulty urinating, frequent urination, and pruritus of vagina); (d) negative for bacterial and fungal culture. This study was approved by the Ethics Committee of Meizhou People's Hospital (Reference No.: MPH‐HEC 2013‐A‐01). Written informed consent was obtained from all participants.

### 2.2 Specimen collection

Cervical and urethral swabs (Kangjian Medical, Jiangsu, China) were used to collected samples from urogenital tracts. In female patients, cervical samples were obtained by cervical swabs from the cervix area after cleaning the exocervical mucus. In male patients, urethral samples were slowly taken from urethra inside 2 cm after external meatus had been cleaned; semen and prostatic fluid were collected and placed in a sterile cup (Kangjian Medical, Jiangsu, China). All samples were sent at room temperature to the clinical laboratory for examination within 2 hour.

### 2.3 Culture and antimicrobial susceptibility test of *Uu* and *Mh*

The culture and susceptibility testing of *Uu* and *Mh* were performed using Mycoplasma ID/AST kit (DL medical), following the manufacture's protocol. Briefly, the specimen swab was inserted into medium flask and mixed intensively to make sample completely dissolve. Then transfer 100 μL of mixture medium to the mycoplasma ID/AST strip. The negative control was added with 100 μL medium. Then the wells were added two drops of the sterile mineral oil and inoculated at 37°C. The results were checked at 24 hours and 48 hours after incubation. The concentration of each organism and resistance or susceptibility to drug was determined on the color change. The susceptibility of mycoplasma toward twelve antibiotics were tested, including tetracycline (TET), levofloxacin (LEV), erythromycin (ERY), josamycin (JOS), doxycycline (DOX), ciprofloxacin (CIP), ofloxacin (OFX), minocycline (MIN), roxithromycin (ROX), azithromycin (AZM), clarithromycin (CLR), and sparfloxacin (SPA).

### 2.4 Statistical analysis

Data are presented as n (%) prevalence or mean ± SD and were statistically analyzed using SPSS 20.0 software (IBM Corp., Armonk, NY, USA). Quantitative data were analyzed using ANOVA test, and categorical data were analyzed using chi‐squared test. *P* < .05 was considered statistically significant.

## 3 RESULTS

### 3.1 Prevalence of *Uu* and *Mh*

Of the 16 118 individuals, 170 patients (112 non‐Hakka and 58 under 16 years of age) were excluded in this study as they did not meet the inclusion criteria. Finally, 15 948 participants were enrolled in this study for analysis, including 12 633 females, aged 34.0 ± 10.0 years, and 3315 males, aged 35.0 ± 11.0 years. Of these females, 5049 (40.0%) were positive for mycoplasma (included *Uu*, *Mh* and *Uu* & *Mh* infection). Among them, 4038 (32.0%) were positive for *Uu*, 135 (1.0%) for *Mh* and 876 (7.0%) for the both. Of clinical samples from males, 748 (22.6%) were positive for mycoplasma. Among them, 660 (20.0%) were positive for *Uu*, 17 (0.5%) for *Mh*, and 71 (2.1%) for the both. The positive rates of all three patterns of infection were significantly higher in females than in males, respectively (*P* < .05).

**Figure 1:**
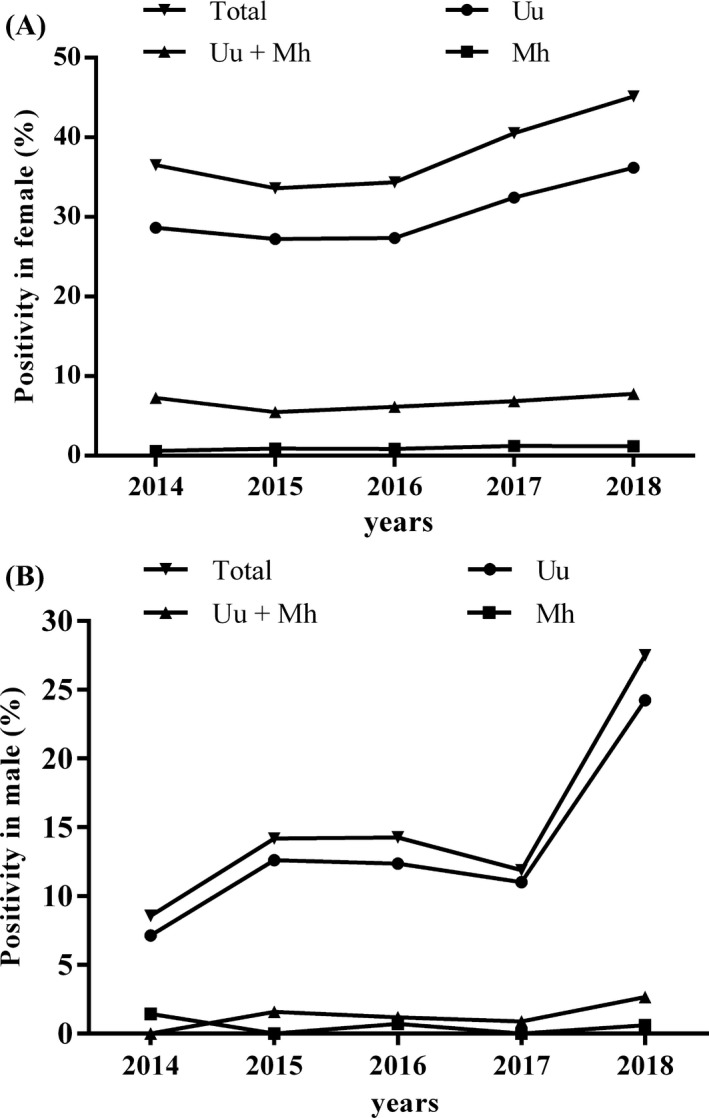
Trend of the prevalence of *Uu* and *Mh* in female and male patients

As shown in Figure 1, the total incidence as well as *Uu* infection was significantly increased in both women and men from 2014 to 2018 (*P* < .05). The positive rates of *Mh* or *Uu* &*Mh* infection were relatively stabilized in both women and men from 2014 to 2018 (*P* > .05).

### 3.2 Age distribution of mycoplasma infection

**Figure 2:**
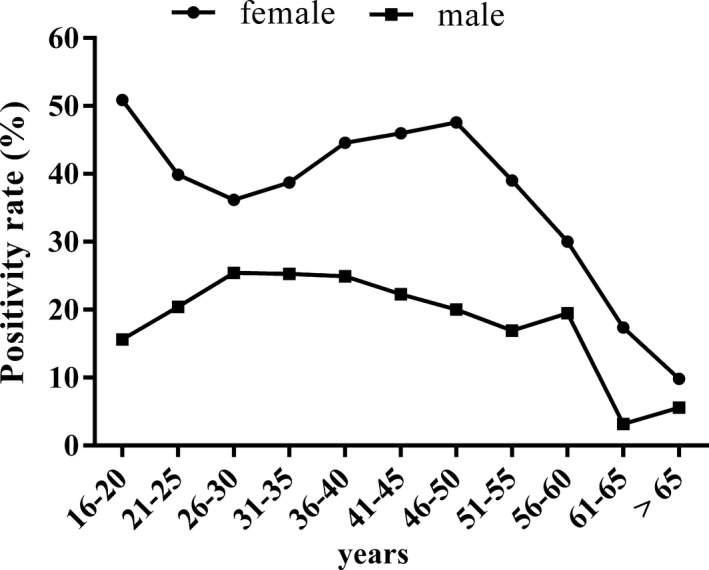
Age‐specific prevalence of *Uu* and *Mh* in female and male patients

The Figure 2 presents an age‐specific mycoplasma infection in Hakka population. As for female participants, the positive rates remained high in the age group of 16‐50 and began to go down gradually more than 50 year. To be noted, the infection rates were relatively high in the age groups of 16‐20 and 36‐50. Of the 3315 males enrolled, the positive rate was stable in the age group of 16‐60, and dramatically dropped after 60 years. Comparatively, the occurrence rate of mycoplasma infection was obviously higher in female than male in all of the age groups.

### 3.3 Antimicrobial susceptibility patterns from 2014 to 2018

Our study found that *Uu* displayed low resistance rates to TET, LEV, ERY, JOS, DOX, OFX, MIN, ROX, AZM, CLR, and SPA, while relatively high resistance rates to CIP (>60%), as shown in Table 1. *Mh* displayed low resistance rates to TET, LEV, JOS, DOX, CIP, OFX, MIN, and SPA, while high resistance rates to ERY (>80%), ROX (>90%), AZM (>90%), and CLR (>85%; Table 2). Furthermore, *Uu* & *Mh* infection exhibited low resistance rates to TET, LEV, JOS, DOX, OFX, MIN, and SPA, but much higher to ERY (>95%), CIP (>85%), ROX (>90%), AZM (>90%), and CLR (>90%; Table 3). Increasing trend was not observed in resistance rates of three infection patterns to antibacterial agents from 2014 to 2018.

**Table 1:** Antimicrobial resistance rate (%) of *Uu* infection in patients

	2014	2015	2016	2017	2018
	Case (%)	Case (%)	Case (%)	Case (%)	Case (%)
TET	12 (6.1)	37 (6.9)	39 (6.0)	44 (11.9)	176 (6.9)
LEV	12 (6.1)	31 (5.8)	19 (2.9)	34 (4.5)	161 (6.3)
ERY	25 (12.6)	128 (23.9)	87 (13.4)	164 (21.5)	267 (10.5)
JOS	1 (0.5)	2 (0.4)	0 (0.0)	0 (0.0)	5 (0.2)
DOX	9 (4.5)	26 (4.9)	22 (3.4)	25 (3.3)	117 (4.6)
CIP	126 (63.6)	396 (73.9)	480 (73.8)	589 (77.1)	1881 (73.8)
OFX	11 (5.6)	27 (5.0)	21 (3.2)	44 (5.8)	89 (3.5)
MIN	9 (4.5)	25 (4.7)	21 (3.2)	25 (3.3)	104 (4.1)
ROX	12 (6.1)	53 (9.9)	29 (4.5)	86 (11.3)	114 (4.5)
AZM	10 (5.1)	32 (6.0)	10 (1.5)	26 (3.4)	58 (2.3)
CLR	9 (4.5)	26 (4.9)	11 (1.7)	25 (3.3)	54 (2.1)
SPA	15 (7.6)	89 (16.6)	76 (11.7)	195 (25.5)	261 (10.2)
n	198	536	650	764	2550

Abbreviations: AZM, azithromycin; CIP, ciprofloxacin; CLR, clarithromycin; DOX, doxycycline; ERY, erythromycin; JOS, josamycin; LEV, levofloxacin; MIN, minocycline; OFX, ofloxacin; ROX, roxithromycin; SPA, sparfloxacin; TET, tetracycline; *Uu, Ureaplasma urealyticum*.

**Table 2:** Antimicrobial resistance rate (%) of *Mh* infection in patients

	2014	2015	2016	2017	2018
	Case (%)	Case (%)	Case (%)	Case (%)	Case (%)
TET	0 (0.0)	2 (12.5)	3 (13.6)	1 (3.4)	14 (17.5)
LEV	2 (40.0)	5 (31.3)	8 (36.4)	9 (31.0)	39 (48.8)
ERY	5 (100.0)	15 (93.8)	20 (90.9)	28 (96.6)	65 (81.3)
JOS	0 (0.0)	0 (0.0)	0 (0.0)	0 (0.0)	1 (1.3)
DOX	0 (0.0)	0 (0.0)	0 (0.0)	0 (0.0)	0 (0.0)
CIP	3 (60.0)	9 (56.3)	11 (50.0)	13 (44.8)	48 (60.0)
OFX	1 (20.0)	6 (37.5)	7 (31.8)	9 (31.0)	31 (38.8)
MIN	0 (0.0)	0 (0.0)	1 (4.5)	0 (0.0)	2 (2.5)
ROX	5 (100.0)	16 (100.0)	20 (90.9)	28 (96.6)	73 (91.3)
AZM	5 (100.0)	16 (100.0)	20 (90.9)	28 (96.6)	73 (91.3)
CLR	5 (100.0)	15 (93.8)	21 (95.5)	28 (96.6)	69 (86.3)
SPA	2 (40.0)	6 (37.5)	7 (31.8)	8 (27.6)	25 (31.3)
n	5	16	22	29	80

Abbreviations: AZM, azithromycin; CIP, ciprofloxacin; CLR, clarithromycin; DOX, doxycycline; ERY, erythromycin; JOS, josamycin; LEV, levofloxacin; *Mh, Mycoplasma hominis*; MIN, minocycline; OFX, ofloxacin; ROX, roxithromycin; SPA, sparfloxacin; TET, tetracycline.

**Table 3:** Antimicrobial resistance rate (%) of *Uu* and *Mh* co‐infection in patients

	2014	2015	2016	2017	2018
	Case (%)	Case (%)	Case (%)	Case (%)	Case (%)
TET	11 (22.4)	30 (28.6)	32 (23.0)	46 (28.6)	119 (24.1)
LEV	28 (57.1)	56 (53.3)	77 (55.4)	102 (63.4)	295 (59.8)
ERY	49 (100)	102 (97.1)	136 (97.8)	154 (95.7)	490 (99.4)
JOS	0 (0.0)	8 (7.6)	5 (3.6)	2 (1.2)	5 (1.0)
DOX	3 (6.1)	6 (5.7)	9 (6.5)	11 (6.8)	34 (6.9)
CIP	42 (85.7)	95 (90.5)	127 (91.4)	152 (94.4)	452 (91.7)
OFX	25 (51.0)	59 (56.2)	73 (52.5)	98 (60.9)	260 (52.7)
MIN	2 (4.1)	3 (2.9)	11 (7.9)	12 (7.5)	38 (7.7)
ROX	48 (98.0)	99 (94.3)	131 (94.2)	150 (93.2)	462 (93.7)
AZM	48 (98.0)	97 (92.4)	127 (91.4)	148 (91.9)	456 (92.5)
CLR	48 (98.0)	98 (93.3)	129 (92.8)	148 (91.9)	455 (92.3)
SPA	27 (55.1)	67 (63.8)	89 (64.0)	110 (68.3)	237 (48.1)
n	49	105	139	161	493

Abbreviations: AZM, azithromycin; CIP, ciprofloxacin; CLR, clarithromycin; DOX, doxycycline; ERY, erythromycin; JOS, josamycin; LEV, levofloxacin; *Mh, Mycoplasma hominis*; MIN, minocycline; OFX, ofloxacin; ROX, roxithromycin; SPA, sparfloxacin; TET, tetracycline; *Uu, Ureaplasma urealyticum*.

As shown in Table 4, the drug resistance was also different between female and male patients. The mycoplasma, either *Uu* or *Mh*, that infected females presented higher resistance to TET, LEV, ERY, DOX, OFX, ROX, AZM, CLR, and SPA, as compared with those infected males.

**Table 4:** Antimicrobial resistance rate (%) of *Uu* and *Mh* infection in patients

	*Uu*		*Mh*		*Uu* & *Mh*	
	Female	Male	Female	Male	Female	Male
TET	271 (6.7)	37 (5.6)	19 (14.1)	1 (5.9)	223 (25.5)	15 (21.1)
LEV	233 (5.8)	24 (3.6)*	60 (44.4)	3 (17.6)*	527 (60.2)	31 (43.7)*
ERY	629 (15.6)	42 (6.4)*	123 (91.1)	10 (58.8)*	850 (97.0)	61 (85.9)*
JOS	6 (0.2)	2 (0.3)	0 (0.0)	1 (5.9)	17 (1.9)	3 (4.2)
DOX	174 (4.3)	25 (3.8)	0 (0.0)	0 (0.0)	56 (6.4)	7 (9.9)
CIP	3046 (75.4)	426 (64.6)*	78 (57.8)	6 (35.3)*	809 (92.4)	59 (83.1)*
OFX	178 (4.4)	14 (2.1)*	53 (39.3)	1 (5.9)*	492 (56.2)	23 (32.4)*
MIN	167 (4.1)	17 (2.6)	3 (2.2)	0 (0.0)	62 (7.1)	4 (5.6)
ROX	265 (6.6)	29 (4.4)*	130 (96.3)	12 (70.6)*	832 (95.0)	58 (81.7)*
AZM	125 (3.1)	11 (1.7)*	129 (95.6)	13 (76.5)*	816 (93.2)	60 (84.5)*
CLR	116 (2.9)	9 (1.4)*	125 (92.6)	13 (76.5)*	818 (93.4)	60 (84.5)*
SPA	616 (15.3)	20 (3.0)*	47 (34.8)	1 (5.9)*	517 (59.0)	13 (18.3)*
n	4038	660	135	17	876	71

Abbreviations: AZM, azithromycin; CIP, ciprofloxacin; CLR, clarithromycin; DOX, doxycycline; ERY, erythromycin; JOS, josamycin; LEV, levofloxacin; *Mh, Mycoplasma hominis*; MIN, minocycline; OFX, ofloxacin; ROX, roxithromycin; SPA, sparfloxacin; TET, tetracycline; *Uu, Ureaplasma urealyticum*.

* *P* < .05.

## 4 DISCUSSION

To our knowledge, this is the first large‐scale study to investigate the prevalence of mycoplasmas infection and antimicrobial resistance in patients with genital symptoms among Hakka population in Meizhou.

The present study suggested that the incidence of mycoplasmas was 40.0% in females, which is lower than the previous findings in other areas of China,^10^, ^11^ but higher than those in Italy.^12^, ^13^ Some reasons may contribute to the variety. First, physical state (symptomatic, asymptomatic, and pregnant) of participants, as well as the socioeconomic conditions varied in different studies. Second, these studies used different sample types and sample size that may draw different conclusion.

Our study showed that the incidence of mycoplasmas infection in females increased annually during the past some years, which was similar to the results in Changzhou, China.^14^ Specifically, positive rate of *Uu* infection kept up‐going in the past five years, while that of *Mh* and *Uu* & *Mh* infection remained stable. The increasing positive rate in our study may be caused by several reasons. On one hand, we stopped testing urethral swab specimens since 2018, which was also a regular type of genital sample but proved much less positive rate for mycoplasmas infection. On the other hand, more and more people accepted genitourinary tests with the improved living condition and implement of national universal two‐child policy after 2016. However, as previous studies suggested, mycoplasmas infection may not cause diseases, but remained as a normal symbiotic colonization. In the present study, we investigated the prevalence of mycoplasmas in patients who showed urogenital symptoms and were excluded from other bacterial or fungal infections. The high positive rate in these samples highlighted the necessity of mycoplasmas examination, thus to facilitate the diagnosis and develop treatment strategy.

Previous research suggested that the infection level of *Uu* and *Mh* was associated with gender and age.^15^ In the current study, positive rates of three patterns of infection in females were much higher than in males. For one thing, the structure and environment of female genital system is more susceptible for the colonization of mycoplasmas. For another, the cervical specimen had the highest positive rate as reported in other studies.^13^

Our study found that in Hakka population, the mycoplasmas infection rates peaked in individuals at the age groups of 16‐20 and 36‐50, and dramatically dropped in patients older than 55. The age‐specific infection were similar to other studies.^7^, ^16^, ^17^ The reduced sexual activity of menopausal women may explained the decreased mycoplasmas infections in elderly people.

As for antimicrobial resistance, resistance rates of *Uu* infection to CIP and resistance rates of *Mh* infection to ERY, ROX, AZM, and CLR, as well as resistance rates of *Uu* & *Mh* infection to ERY, CIP, ROX, AZM, and CLR were higher, which were in consistence with results reported in other areas.^18^, ^19^ Interestingly, resistance rates of *Uu*, *Mh* and *Uu* & *Mh* infection to LEV, ERY, CIP, OFX, ROX, AZM, CLR, and SPA varied between females and males in our study. There are some reasons for this finding. First, female patients infected with mycoplasma were several times more than male patients; Second, female patients generally received more frequent antibiotic treatment in our study, and antibiotic selection pressures were different between females and males. Taking together, *Uu* and *Mh* species tested in our study subjects displayed relatively low resistance to TET, JOS, DOX, and MIN. Since TET caused severe side effects, JOS and MIN may be the main choice for *Uu* and *Mh* in Hakka population.

Although some novel findings were revealed in this study, there are some limitations needed to be clarified. First, the information of sexual behavior of the enrolled subjects was missing, which may partially influence the conclusion. Second, we did not collect lifestyle information thus could not confirm the role of lifestyle on mycoplasmas infection.

In conclusion, our study for the first time investigated the prevalence of mycoplasmas infection as well as drug resistance in Hakka population. Our data revealed the gender‐ and age‐specific infection distribution and the year‐by‐year positive rates. The findings added knowledge to local epidemiology of mycoplasmas and would be useful for prevention and treatment strategy.

## **AUTHORS**'** CONTRIBUTIONS**

ZZ conceived and designed the experiments; XG contributed to the data collection and the manuscript draft. SL, XG, and RW helped to collect clinical data and conducted the clinical performances and researches; XG analyzed the data and wrote the paper.
